# Emerging Biomarkers for the Early Detection of Autoimmune Encephalitis: A Narrative Review

**DOI:** 10.7759/cureus.69038

**Published:** 2024-09-09

**Authors:** Syed Faqeer Hussain Bokhari, Mohammed Khaleel I.KH. Almadhoun, Muhammad U Khan, Shahzad Ahmad, Manahil Awan, Muhammad Mustafa Imran, Muhammad Bashir, Muhammad Rohail Tariq, Minahil Imran, Muhammad Omer Khalid

**Affiliations:** 1 Medicine and Surgery, King Edward Medical University, Lahore, PAK; 2 Medicine and Surgery, Mutah University, Karak, JOR; 3 Cardiac Surgery, Liaquat National Hospital, Karachi, PAK; 4 Executive and Special Ward, Liaquat National Hospital, Karachi, PAK; 5 Medicine and Surgery, Jinnah Medical and Dental College, Karachi, PAK

**Keywords:** autoantibodies, autoimmune encephalitis, biomarkers, metabolomics, neurofilament light chain, neuroimaging, proteomics

## Abstract

Autoimmune encephalitis (AE) is a rare yet critical neurological disorder characterized by inflammation of the brain, typically triggered by an abnormal immune response. The early detection and diagnosis of AE are crucial for effective treatment and improved patient outcomes. However, the diagnostic process is often complicated by the diverse clinical presentations of AE, which can mimic other neurological and psychiatric conditions. Currently, diagnosis relies on a combination of clinical evaluation, neuroimaging, cerebrospinal fluid analysis, and the detection of specific autoantibodies. Despite advances in these areas, challenges remain, particularly in cases where patients are seronegative or present with nonspecific symptoms. This narrative review provides a comprehensive overview of emerging biomarkers for the early detection of AE, highlighting their potential to enhance diagnostic accuracy and speed. We explore a variety of biomarkers, including novel autoantibodies, inflammatory markers, cytokines, and neuronal damage indicators, and discuss their clinical implications. This review emphasizes the need for biomarkers that are not only sensitive and specific but also accessible and rapid to facilitate earlier diagnosis and treatment. By synthesizing current research, this review aims to contribute to the ongoing efforts to refine the diagnostic approach to AE, ultimately improving outcomes for patients affected by this challenging condition.

## Introduction and background

Autoimmune encephalitis (AE) represents a group of rare but potentially devastating neurological disorders characterized by inflammation of the brain due to an aberrant immune response [1]. This condition has gained significant attention in recent years due to its complex presentation and the critical importance of early diagnosis and treatment. AE can affect individuals of all ages, though certain subtypes show a predilection for specific age groups [1,2]. The clinical manifestations of AE are diverse and can include a wide range of neuropsychiatric symptoms, such as memory deficits, behavioral changes, seizures, and altered mental status. These varied presentations often lead to diagnostic challenges, as the symptoms can mimic other neurological and psychiatric conditions.

The importance of early detection in AE cannot be overstated. Timely diagnosis and initiation of appropriate immunotherapy are crucial factors in improving patient outcomes, reducing the risk of long-term neurological sequelae, and enhancing overall quality of life. However, the path to diagnosis is often fraught with difficulties. The nonspecific nature of initial symptoms, combined with the rarity of the condition, can lead to delays in recognition and treatment [3]. Furthermore, the overlap of AE symptoms with those of infectious encephalitis, primary psychiatric disorders, and other autoimmune conditions adds another layer of complexity to the diagnostic process [4]. In this context, the role of biomarkers in the early detection of AE has emerged as a critical area of research. Biomarkers, defined as objectively measurable indicators of biological processes or responses to therapeutic interventions, have the potential to revolutionize the approach to AE diagnosis. The ideal biomarker for AE would provide high sensitivity and specificity, allowing for rapid and accurate identification of the condition in its early stages. This would enable clinicians to differentiate AE from mimicking disorders and initiate appropriate treatment promptly.

Currently, the diagnosis of AE relies heavily on a combination of clinical presentation, neuroimaging findings, cerebrospinal fluid (CSF) analysis, and the detection of specific autoantibodies [5]. While these methods have significantly improved the ability to diagnose AE, they are not without limitations. Autoantibody testing, for instance, can be time-consuming and may not be universally available. Moreover, a subset of patients with suspected AE remains antibody-negative despite clinical evidence of the disease, highlighting the need for additional diagnostic tools. The development of novel biomarkers for AE addresses several critical gaps in the current diagnostic approach. First, it aims to provide more rapid and accessible diagnostic methods, potentially reducing the time to diagnosis and treatment initiation. Second, new biomarkers could enhance the sensitivity and specificity of AE diagnosis, particularly in cases where current methods yield inconclusive results. Finally, biomarkers may offer insights into disease mechanisms, severity, and prognosis, thereby informing treatment decisions and monitoring response to therapy.

The objective of this narrative review is to provide a comprehensive overview of emerging biomarkers for the early detection of AE. To conduct this narrative review, we performed a comprehensive literature search using major medical databases including PubMed, Embase, and Web of Science. Key search terms included "autoimmune encephalitis," "biomarkers," "diagnosis," "prognosis," and various combinations thereof. We focused on articles published within the last ten years to capture the most recent developments in the field. This multifaceted approach allowed us to compile a thorough overview of the current state of knowledge regarding emerging biomarkers for the early detection of AE.

## Review

Overview

AE is a complex neurological disorder characterized by inflammation of the brain parenchyma due to aberrant immune responses. The pathophysiology of AE involves the production of autoantibodies that target specific neuronal antigens, leading to the disruption of normal neuronal function and signaling. These autoantibodies can be classified into two main categories: those targeting intracellular antigens (e.g., Hu, Ma2, glutamic acid decarboxylase 65 [GAD65]) and those targeting cell-surface or synaptic proteins (e.g., N-methyl-D-aspartate [NMDA] receptor, leucine-rich glioma-inactivated 1 [LGI1], contactin-associated protein-like 2 [CASPR2]) [6]. The NMDA receptor antibody is one of the most well-characterized in AE. It targets the GluN1 subunit of the NMDA receptor, leading to receptor internalization and subsequent glutamatergic dysfunction. This disruption can result in a wide array of neuropsychiatric symptoms. LGI1 and CASPR2 antibodies are often associated with limbic encephalitis, affecting voltage-gated potassium channels and leading to hyperexcitability of neurons [7].

The clinical presentation of AE is notably heterogeneous, often posing significant diagnostic challenges. Common symptoms include psychiatric manifestations such as psychosis, agitation, and mood disturbances; cognitive impairments, particularly short-term memory deficits; and seizures, which can range from focal to generalized [8,9]. The variability in presentation is largely dependent on the underlying antibody and the specific brain regions affected. For instance, anti-NMDA receptor encephalitis often presents with prominent psychiatric symptoms and may progress to autonomic instability and movement disorders. By contrast, LGI1 antibody-associated encephalitis frequently features prominent memory impairment and faciobrachial dystonic seizures [10]. The current diagnostic criteria for AE, as proposed by Graus et al. in 2016, include a combination of clinical features, laboratory findings, and the exclusion of alternative causes [11]. These criteria have improved diagnostic accuracy but still have limitations, particularly in the early stages of the disease when symptoms may be subtle or nonspecific (Table 1).

**Table 1:** Differential diagnosis of autoimmune encephalitis anatomical syndromes and suggested additional testing ADEM, acute disseminated encephalomyelitis; AHL, acute hemorrhagic leukoencephalitis; ANA, antinuclear antibody; AQP4, aquaporin-4; CJD, Creutzfeldt-Jacob disease; CLIPPERS, chronic lymphocytic inflammation with pontine perivascular enhancement responsive to steroids; CPM, central pontine myelinolysis; CSF, cerebrospinal fluid; DaT scan, dopamine transporter scan; ENA, extractable nuclear antigens; GFAP, glial fibrillar acidic protein; GPA, gliomatosis with polyangitis; GQ1b, anti-ganglioside Q1B antibody; HHV6, human herpes virus-6; HSV, herpes simplex virus; JCV, John Cunningham virus; MOG, myelin oligodendrocyte glycoprotein; MS, multiple sclerosis; MSA-C, cerebellar multiple system atrophy; NMOSD, neuromyelitis optica spectrum disorder; PML, progressive multifocal leukoencephalopathy; SCA, spinocerebellar ataxia; VEP, visual evoked potentials; VZV, varicella zoster virus; WNV, West Nile virus Adapted with permission from Abboud et al. [12]

Anatomical classification of autoimmune encephalitis	Differential diagnosis	Possible additional testing as appropriate
Limbic encephalitis	HSV, VZV, HHV6	CSF viral PCR, CSF VZV IgG/IgM
Cortical/subcortical encephalitis	ADEM, AHL, tumefactive MS, Marburg, PML, CJD, lupus cerebritis, Behcet, neurosarcoidosis, neurosyphilis, lymphoma, anoxic injury, seizure-related changes	MOG-IgG, CSF JCV PCR, CSF prion panel (RTQuIC), ANA/ENA, HLA-B51, ACE, CT chest (to rule out sarcoidosis), treponemal antibodies, CSF cytology and flow cytometry
Striatal encephalitis	CJD, WNV, toxic encephalopathy, anoxic injury, hyperglycaemic injury, uraemia	Prion panel, WNV IgM, CSF viral PCR, toxicology screen, metabolic panel
Diencephalic encephalitis	Neurosarcoidosis, Behcet, Wernicke, Whipple	ACE, CT chest (to rule out sarcoidosis), HLA-B51, thiamine level
Brainstem encephalitis	Rhombencephalitis (listeria), viral, CLIPPERS, neurosarcoidosis, Behcet, lymphoma, PML, CPM, Erdheim-Chester, Whipple	CSF bacterial culture, CSF viral PCR, HLA-B51, CSF cytology and flow cytometry, CSF JCV PCR, bone scan
Cerebellitis or cerebellar degeneration	Viral or post-viral cerebellitis, coeliac disease, Miller Fisher syndrome, vitamin E deficiency, MSA-C, SCA	Viral PCR, coeliac antibodies, anti-GQ1b, vitamin E level, DaT scan
Meningoencephalitis	Tuberculosis, neurosarcoidosis, Behcet, bacterial or viral infection, leptomeningeal carcinomatosis, GPA, IgG4-related disease	Bacterial PCR, ACE, CT chest, HLA-B51, CSF bacterial culture, CSF viral PCR, CSF cytology and flow cytometry
Encephalomyelitis and/or opticospinal syndrome	ADEM, WNV	MOG-IgG, WNV IgM, CSF viral PCR

The diagnostic approach to AE involves a multifaceted evaluation. Clinical assessment remains the cornerstone, with a detailed history and neurological examination guiding further investigations. Neuroimaging, particularly magnetic resonance imaging (MRI), plays a crucial role in identifying brain inflammation and excluding other pathologies [13]. However, it is important to note that MRI findings can be normal in a significant proportion of AE cases, especially in the early stages [14]. Cerebrospinal fluid (CSF) analysis is another critical component of the diagnostic workup. Typical findings include mild to moderate pleocytosis, elevated protein levels, and the presence of oligoclonal bands. The detection of specific autoantibodies in CSF and/or serum remains the gold standard for definitive diagnosis of many forms of AE [15,16]. Traditional biomarkers such as oligoclonal bands, while supportive of an autoimmune process, lack specificity for AE.

The use of antibody titers as biomarkers has been valuable but has several limitations. First, the detection of these antibodies can take days to weeks, potentially delaying diagnosis and treatment. Second, not all patients with clinical features of AE have detectable antibodies with current testing methods, leading to the classification of "seronegative" AE [17]. Third, antibody titers may not always correlate well with disease severity or prognosis. Furthermore, the current diagnostic approaches face challenges in differentiating AE from other conditions that can present similarly, such as infectious encephalitis, primary psychiatric disorders, or other autoimmune conditions affecting the central nervous system. This differentiation is crucial for the timely initiation of appropriate treatment. These limitations underscore the need for more rapid, sensitive, and specific biomarkers for the early detection of AE. Emerging biomarkers aim to address these gaps, potentially allowing for earlier diagnosis, more accurate monitoring of disease activity, and better prediction of treatment response and long-term outcomes.

Established biomarkers

Autoantibodies have emerged as the most significant and specific biomarkers in AE. Anti-NMDA receptor encephalitis is now recognized as one of the most common forms of AE [18]. These antibodies target the GluN1 subunit of the NMDA receptor, leading to receptor internalization and subsequent glutamatergic dysfunction. The detection of NMDA receptor antibodies in CSF and/or serum is highly specific for the diagnosis of anti-NMDA receptor encephalitis [19]. Importantly, antibody titers, particularly in CSF, have been shown to correlate with clinical outcomes. Higher titers are often associated with more severe presentations and prolonged recovery times, while a decrease in titers typically corresponds with clinical improvement. However, it is worth noting that some patients may have persistent cognitive deficits even after antibody titers have normalized, highlighting the complex relationship between antibody levels and clinical manifestations [20,21].

LGI1 and contactin-associated protein-like 2 (CASPR2) antibodies are primarily associated with limbic encephalitis, a subtype of AE affecting the limbic system [22-24]. These antibodies target proteins associated with voltage-gated potassium channels, leading to neuronal hyperexcitability. LGI1 antibodies are more commonly associated with limbic encephalitis, while CASPR2 antibodies can cause a broader spectrum of neurological syndromes, including Morvan syndrome and neuromyotonia [23]. The detection of these antibodies is highly specific for their respective syndromes and is crucial for differentiating them from other forms of AE. LGI1 antibody-associated encephalitis often presents with distinctive clinical features, such as faciobrachial dystonic seizures, which can precede the onset of cognitive symptoms [25]. This unique presentation, combined with antibody detection, allows for early diagnosis and treatment initiation.

Gamma-aminobutyric acid B (GABA(B)) receptor and α-amino-3-hydroxy-5-methyl-4-isoxazolepropionic acid (AMPA) receptor antibodies represent another important group of autoantibodies in AE [26]. GABA(B) receptor antibodies are often associated with limbic encephalitis and prominent seizures. These antibodies can be found in both paraneoplastic and non-paraneoplastic cases, with small-cell lung cancer being the most common associated malignancy [27]. AMPA receptor antibodies, on the other hand, target glutamate receptors and are frequently associated with limbic encephalitis. These antibodies are more commonly found in paraneoplastic cases, with lung, breast, and thymic tumors being the most frequently associated malignancies [28]. The detection of GABA(B) and AMPA receptor antibodies is crucial not only for diagnosing the specific subtype of AE but also for guiding the search for underlying malignancies in affected patients.

In addition to autoantibodies, inflammatory markers play a significant role as biomarkers in AE. Cytokines and chemokines, key mediators of the immune response, have been found to be elevated in both CSF and serum of patients with AE. These inflammatory markers can provide valuable information about the underlying immune processes and potentially aid in the diagnosis and monitoring of disease activity. For instance, interleukin-6 (IL-6) levels in CSF have been found to be significantly elevated in patients with anti-NMDA receptor encephalitis [29]. Other cytokines, such as interferon-gamma (IFN-γ), tumor necrosis factor-alpha (TNF-α), and interleukin-17 (IL-17), have also been implicated in various subtypes of AE [29]. The specific cytokine profiles may differ depending on the underlying antibody, potentially offering a means to differentiate between AE subtypes. However, it is important to note that these inflammatory markers are not specific to AE and can be elevated in other inflammatory conditions of the central nervous system, limiting their use as standalone diagnostic tools.

The complement system, a key component of the innate immune response, has also been investigated as a potential biomarker in AE. Complement activation has been demonstrated in several forms of AE, with deposits of complement components found in the brain tissue of affected patients. Elevated levels of complement proteins, such as C3a and C5a, have been detected in the CSF of patients with anti-NMDA receptor encephalitis [30]. While these findings suggest a role for complement activation in the pathogenesis of AE, the utility of complement components as diagnostic markers is still under investigation. Their lack of specificity and the technical challenges in measuring them reliably in clinical settings currently limit their widespread use as biomarkers.

Imaging biomarkers, particularly those derived from MRI, play a crucial role in the diagnosis and monitoring of AE. Typical MRI findings in AE include T2/FLAIR hyperintensities, most commonly affecting the medial temporal lobes in limbic encephalitis [31]. However, the distribution of abnormalities can vary depending on the specific subtype of AE. For instance, anti-NMDA receptor encephalitis may show more diffuse cortical and subcortical T2/FLAIR hyperintensities, while LGI1 antibody-associated encephalitis often presents with unilateral or bilateral medial temporal lobe involvement [31]. It is important to note that MRI findings can be normal in a significant proportion of AE cases, especially in the early stages of the disease. The sensitivity and specificity of MRI findings in AE vary depending on the specific antibody and the timing of the imaging. While certain patterns are suggestive of AE, they are not pathognomonic and can be seen in other conditions, including infectious encephalitis and seizure-related changes. Therefore, MRI findings should always be interpreted in the context of the clinical presentation and other diagnostic tests.

Advanced imaging techniques, such as positron emission tomography (PET) and single-photon emission computed tomography (SPECT), have shown promise in identifying early changes in AE [32]. Fluorodeoxyglucose (FDG)-PET, in particular, can reveal areas of hypermetabolism in AE, even when structural MRI appears normal [33]. These metabolic changes often precede structural abnormalities and can be more extensive than the changes seen on MRI. For example, in anti-NMDA receptor encephalitis, FDG-PET may show frontotemporal hypermetabolism in the early stages, followed by diffuse cerebral hypometabolism in later stages [34]. SPECT imaging, while less commonly used, can also demonstrate perfusion abnormalities in AE [35]. The use of PET and SPECT in AE is still evolving, and these modalities are not routinely used in all clinical settings due to limited availability and higher costs. However, they offer the potential for earlier detection of brain involvement in AE and may provide additional information about disease activity and treatment response (Table 2).

**Table 2:** Pros and cons of established biomarkers in autoimmune encephalitis. AE: autoimmune encephalitis, CSF: cerebrospinal fluid, MRI: magnetic resonance imaging, EEG: electroencephalogram, IL-6: interleukin-6, TNF-α: tumor necrosis factor-alpha, FDG-PET: fluorodeoxyglucose positron emission tomography

Biomarker	Pros	Cons
Autoantibodies (e.g., anti-NMDAR, anti-LGI1, anti-CASPR2)	High specificity for AE subtypes. Directly linked to pathogenesis. Can guide treatment decisions.	Not all patients with AE have detectable antibodies (seronegative cases). Testing can be time-consuming and may not correlate well with disease severity or prognosis in all cases.
CSF analysis (pleocytosis, protein levels, oligoclonal bands)	Widely available and can support the diagnosis of the inflammatory process	Low specificity for AE. Can be normal in some AE cases. Requires invasive lumbar puncture
MRI findings	Non-invasive and can exclude other pathologies. Specific patterns can suggest AE.	Can be normal in early stages or some AE subtypes. Findings can be non-specific Limited sensitivity in some AE cases.
EEG changes	Can detect subclinical seizures. Specific patterns (e.g., extreme delta brush in anti-NMDAR encephalitis) can be suggestive.	Low specificity for AE. Requires expertise for interpretation. Findings can be variable and non-specific.
Inflammatory markers (e.g., IL-6, TNF-α)	Can indicate ongoing inflammation and may correlate with disease activity	Low specificity for AE. Can be elevated in various inflammatory conditions. Limited availability of testing in some settings
FDG-PET	Can show abnormalities before structural MRI changes and may help in monitoring the treatment response	Limited availability and high-cost exposure to radiation. Patterns can overlap with other conditions.

Emerging biomarkers

Novel autoantibodies continue to be discovered, expanding our understanding of AE and providing new diagnostic tools. One such antibody is directed against dipeptidyl-peptidase-like protein 6 (DPPX), a regulatory subunit of the Kv4.2 potassium channel [36]. DPPX antibodies are associated with a distinct clinical syndrome characterized by gastrointestinal hyperexcitability, central nervous system hyperexcitability, and cognitive impairment [37]. The discovery of DPPX antibodies has significant diagnostic implications, as it allows for the identification of a previously unrecognized form of AE. Patients with DPPX antibodies often present with prominent gastrointestinal symptoms preceding neurological manifestations, which can lead to initial misdiagnosis [38]. The recognition of this antibody has thus expanded the clinical spectrum of AE and highlighted the importance of considering AE in patients with multisystem involvement.

Another important emerging antibody is directed against IgLON5, a neuronal cell adhesion protein. IgLON5 antibodies were first reported in 2014 and are associated with a complex neurological syndrome that includes sleep disorders, gait instability, bulbar symptoms, and cognitive decline [39]. Interestingly, IgLON5-associated encephalopathy presents with features of both autoimmunity and neurodegeneration, blurring the lines between these two categories of neurological disorders [40]. The presence of IgLON5 antibodies in the context of neurodegenerative features raises intriguing questions about the potential role of autoimmunity in some neurodegenerative conditions. From a diagnostic perspective, testing for IgLON5 antibodies should be considered in patients with atypical neurodegenerative presentations, particularly those with prominent sleep disorders. Another example is antibodies targeting neurexin-3α, which have been linked to a form of AE characterized by seizures and confusion [41]. While these newer antibodies are still in the early stages of characterization, they represent potential future diagnostic tools and may help identify previously unrecognized forms of AE.

Neurofilament light chain (NfL) has emerged as a promising biomarker of neuronal damage across various neurological disorders, including AE. NfL is a structural protein found in neurons, and its release into CSF and blood is indicative of axonal injury [42]. In the context of AE, elevated levels of NfL have been observed in both CSF and serum of patients, correlating with disease severity and clinical outcomes. A study by Mariotto et al. found that serum NfL levels were significantly increased in patients with AE compared to controls and other neurological disorders [43]. Importantly, NfL levels showed a strong correlation with functional outcomes and cognitive performance. The utility of NfL in early diagnosis lies in its high sensitivity to neuronal damage, potentially allowing for the detection of AE before the onset of significant clinical symptoms. Additionally, as a marker of neuronal injury, NfL could provide valuable information about disease activity and treatment response, complementing antibody testing and clinical assessment [43-45] (Table 3).

**Table 3:** Summary of key emerging biomarkers in autoimmune encephalitis AE: autoimmune encephalitis, DPPX: dipeptidyl-peptidase-like protein 6, IgLON5: immunoglobulin-like cell adhesion molecule 5, NfL: neurofilament light chain, MRI: magnetic resonance imaging, fMRI: functional magnetic resonance imaging, DTI: diffusion tensor imaging, IL-6: interleukin-6, IFN-γ: interferon-gamma, TNF-α: tumor necrosis factor-alpha

Biomarker type	Examples	Potential clinical utility
Novel autoantibodies	DPPX, IgLON5, neurexin-3α	Identification of new AE subtypes
Neuronal damage markers	Neurofilament light chain (NfL)	Disease severity, prognosis
Advanced imaging techniques	Quantitative MRI, fMRI, DTI	Early detection of brain changes
Proteomic markers	Chitinase 3-like 1, complement factors	Disease mechanisms, potential therapeutic targets
Metabolomic profiles	Alterations in metabolic pathways	Early detection, insights into pathophysiology
Cytokines/chemokines	IL-6, IFN-γ, TNF-α	Inflammatory status, disease activity

Proteomic and metabolomic approaches offer the potential to identify novel biomarkers and provide insights into the pathophysiology of AE. Proteomic studies, utilizing mass spectrometry techniques, have been employed to analyze the protein composition of CSF in AE patients [46,47]. These studies have identified differential expression of proteins involved in inflammation, synaptic function, and neuronal survival. For example, proteomic analysis has identified several proteins, such as chitinase 3-like 1 and complement factors, as potential biomarkers in anti-NMDA receptor encephalitis [48]. These proteomic signatures could potentially aid in early diagnosis and provide targets for therapeutic intervention.

Metabolomics, the study of small molecule metabolites, is another emerging field with potential applications in AE biomarker discovery. Metabolomic profiling of CSF and serum can reveal alterations in metabolic pathways associated with AE [49,50]. Ge et al. identified distinct cerebral 18F-FDG PET metabolic patterns in anti-NMDAR encephalitis patients, varying by etiological subgroups, with cryptogenic cases showing asymmetric hypermetabolism in frontal-temporal lobes, paraneoplastic cases presenting with symmetric hypermetabolism, and viral encephalitis-related cases exhibiting focal hypometabolism in the affected regions, offering potential insights for early diagnosis and tailored treatment strategies [51]. While metabolomic studies in AE are still limited, this approach holds promise for identifying metabolic biomarkers that could aid in early detection and provide insights into disease mechanisms.

Advanced imaging techniques are pushing the boundaries of early detection in AE. Quantitative MRI techniques, such as volumetric analysis and diffusion tensor imaging (DTI), offer the potential to detect subtle structural changes before they become apparent on conventional MRI [52]. Volumetric analysis can reveal early atrophy patterns, while DTI can detect microstructural white matter changes [53,54]. These advanced MRI techniques could potentially identify patients with AE at earlier stages, before the development of overt structural abnormalities. Functional MRI (fMRI) and connectivity studies provide insights into the functional changes occurring in AE, potentially before the onset of clinical symptoms. Resting-state fMRI studies have shown altered functional connectivity patterns in patients with AE, particularly affecting networks involved in memory and cognition. Studies have demonstrated disrupted functional connectivity in the default mode network in patients with anti-LGI1 encephalitis, correlating with memory deficits [55,56]. These functional changes may precede structural alterations and could serve as early markers of AE. The integration of advanced imaging techniques with other biomarkers, such as autoantibodies and NfL, could significantly enhance our ability to detect and monitor AE in its early stages. For instance, combining functional connectivity data with serum NfL levels might provide a more comprehensive assessment of disease activity and neuronal injury.

Clinical implications and challenges

The translation of emerging biomarkers for AE into clinical practice offers promising improvements in patient care but also introduces significant challenges. Validation and implementation of new biomarkers require rigorous studies to confirm their reliability, sensitivity, and specificity. Initially identified in small-scale studies, these biomarkers must be validated in larger, diverse populations through multi-center studies to account for patient demographics, clinical presentations, and laboratory variations. Once validated, standardization of testing methods is crucial, involving the development of reliable assays, establishing reference ranges, and determining appropriate cut-off values for clinical decision-making. Integrating new biomarkers into current diagnostic workflows offers the potential to enhance early detection and improve diagnostic accuracy. However, their introduction must be managed carefully to avoid disrupting established clinical pathways. This may involve creating new diagnostic algorithms that incorporate both traditional and emerging biomarkers, potentially using a tiered approach where specialized tests follow initial screenings. Clinicians must also be educated on the interpretation and significance of these new biomarkers to ensure their appropriate use.

One of the primary challenges in biomarker development for AE is the inherent variability in biomarker expression among patients. AE encompasses a heterogeneous group of disorders with diverse clinical presentations and underlying pathophysiologies, leading to significant variations in biomarker levels. This variability necessitates large-scale studies with well-characterized patient cohorts to establish reliable reference ranges and understand the factors influencing biomarker levels. The logistical and financial challenges of large-scale studies are compounded by the rarity of AE, requiring multi-center collaborations to achieve adequate sample sizes. Additionally, longitudinal studies are essential to understanding changes in biomarker levels over time and in response to treatment, though these require significant resources. Ethical considerations also arise, particularly in the collection of invasive biological samples like cerebrospinal fluid, and the disclosure of prognostic information to patients.

Despite these challenges, the development of biomarkers for AE holds great promise for advancing personalized medicine. Improved understanding of specific biomarkers and their relationship to disease mechanisms could enable tailored treatment strategies. Biomarker-driven decision-making in therapy selection and monitoring offers the potential for more precise and effective management of AE, balancing treatment efficacy with the minimization of side effects.

Future directions

The rapidly advancing field of AE biomarker research faces significant challenges, particularly the need for comprehensive longitudinal studies. Such studies are essential to track biomarker changes throughout the progression of the disease, from the initial onset through treatment and follow-up. Longitudinal data would offer valuable insights into the natural history of AE and help identify biomarkers predictive of disease progression, relapse risk, and long-term outcomes. In addition, identifying biomarkers that predict therapy response is a critical research priority. Currently, treatment decisions in AE rely on clinical presentation and specific autoantibodies, but patient responses to immunotherapy can vary significantly, even among those with similar antibody profiles. Developing predictive biomarkers could improve patient care by identifying which patients are likely to respond to first-line treatments versus those needing more aggressive or alternative therapies.

Technological advances, including artificial intelligence (AI) and machine learning (ML), are becoming increasingly important in AE biomarker discovery and validation. These tools can analyze complex datasets to identify novel biomarkers or combinations with high diagnostic or prognostic value. The future of AE research is particularly promising with multi-omics approaches that integrate data from genomics, proteomics, and metabolomics. Advanced imaging techniques, such as high-resolution MRI and PET imaging, also hold the potential to identify subtle brain changes in early-stage AE. International collaboration and large-scale, multicenter studies are crucial for validating potential biomarkers across diverse patient populations, emphasizing the importance of consortia and standardized protocols. Future directions should focus on translational research to bridge the gap between basic science discoveries and clinical applications, including developing clinically applicable assays for biomarkers and conducting trials to assess biomarker-guided treatments. As the understanding of AE pathophysiology grows, there may be opportunities for novel therapeutic approaches tailored to specific molecular subtypes of AE, ultimately moving the field closer to personalized, precision medicine for AE patients.

## Conclusions

The field of AE biomarker research is rapidly evolving, offering promising avenues for improving early detection, diagnosis, and patient management. Emerging biomarkers, including novel autoantibodies, neuronal damage indicators like NfL, and advanced imaging techniques, have the potential to address current diagnostic challenges and provide insights into disease mechanisms. However, significant hurdles remain in translating these findings into clinical practice. Rigorous validation studies, standardization of testing methods, and careful integration into existing diagnostic workflows are essential steps in this process. Future research should focus on longitudinal studies to understand biomarker dynamics throughout the disease course, as well as the development of predictive biomarkers for treatment response. The application of artificial intelligence, multi-omics approaches, and advanced imaging techniques holds great promise for identifying new biomarkers and improving diagnostic accuracy. International collaboration and large-scale studies will be crucial for validating these biomarkers across diverse populations.
